# Long-term outcomes and healthcare utilization following critical illness – a population-based study

**DOI:** 10.1186/s13054-016-1248-y

**Published:** 2016-03-31

**Authors:** A. D. Hill, R. A. Fowler, R. Pinto, M. S. Herridge, B. H. Cuthbertson, D. C. Scales

**Affiliations:** Department of Critical Care Medicine, Sunnybrook Health Sciences Centre, Toronto, ON Canada; Sunnybrook Research Institute, Toronto, ON Canada; Interdepartmental Division of Critical Care, University of Toronto, Toronto, ON Canada; Toronto General Hospital/University Health Network, Toronto, ON Canada

**Keywords:** Critical care, Outcomes, Healthcare utilization

## Abstract

**Background:**

The purpose of this study was to examine hospital mortality, long-term mortality, and health service utilization among critically ill patients. We also determined whether these outcomes differed according to demographic and clinical characteristics.

**Methods:**

We conducted a retrospective cohort study of adults (age ≥18 years) who survived admission to an intensive care unit (ICU) in Ontario, Canada, between 1 April 2002 and 31 March 2012, excluding isolated admissions to step-down or intermediate ICUs, coronary care ICUs, or cardiac surgery ICUs. Adults (age ≥18 years) who survived an acute hospitalization that did not include an ICU stay formed the comparator group. The primary outcome was mortality following hospital discharge. Secondary outcomes were healthcare utilization, including emergency room admissions and hospital readmissions during follow-up.

**Results:**

Over the study interval, 500,124 patients were admitted to ICUs and 420,187 (84 %) survived to hospital discharge. Median follow-up for survivors was 5.3 (interquartile range 2.5, 8.2) years. Patients admitted to an ICU were more likely to subsequently visit the emergency department, be readmitted to the hospital and ICU, receive home care support, require rehabilitation, and be admitted for long-term care. Those requiring more resources within the ICU required more resources after discharge. One-third of patients admitted to the ICU died during long-term follow-up, with overall probabilities of death of 11 % and 29 % at 1 year and 5 years, respectively. In the adjusted analysis, there was an increasing hazard of death with increasing age, reaching a hazard ratio of 18.08 (95 % confidence interval 16.60–19.68) for those ≥85 years of age compared with those aged 18–24 years.

**Conclusions:**

Healthcare utilization after hospital discharge was higher among ICU patients, and also among those requiring more healthcare resources during their ICU admission, than among all hospitalized patients as a group. One-third of ICU patients died within the 5 years following discharge, and age was the most influential determinant of outcome. These findings should help target post–ICU discharge services for high-risk groups and better inform goals-of-care discussions for elderly critically ill patients.

**Electronic supplementary material:**

The online version of this article (doi:10.1186/s13054-016-1248-y) contains supplementary material, which is available to authorized users.

## Background

The intensive care unit (ICU) provides potentially life-sustaining interventions to critically ill patients [1, 2]. However, individuals surviving the acute phase of their critical illness often have persistent cognitive, physical, and functional impairment [3–5]. These longer-term effects of critical illness may impact these patients’ continued healthcare needs and their health services utilization [6, 7]. Prior studies in which researchers have examined healthcare use and outcomes among ICU survivors have largely been restricted to specific patient populations, have measured only limited indicators of healthcare use, or have had relatively short follow-up [6–14]. More recently, others have suggested significant post-ICU healthcare utilization among ICU survivors [15]. We conducted a population-based cohort study to examine long-term outcomes and health services utilization among critically ill patients and to determine the extent to which these are influenced by patient characteristics and course of illness.

## Methods

### Patient population and data source

We used the Canadian Institute for Health Information (CIHI) Discharge Abstract Database (DAD) to identify all patients (≥18 years of age) with a hospitalization that included an ICU admission in Ontario, Canada, between 1 April 2002 and 31 March 2012. The DAD includes demographic, clinical, and procedural information on all admissions to acute care hospitals for all Canadian provinces except Quebec. An index admission to the ICU was identified using special care unit codes that identify all admissions to general and specialty care ICUs and have been shown to have high accuracy [16, 17]. The study time frame was chosen to reflect all years with available data following the adoption of the International Classification of Diseases, Tenth Revision (ICD-10), system by CIHI, and to provide at least 1 year of follow-up data for all patients (until 31 March 2013). For patients with multiple hospital episodes that included an ICU admission, we considered the primary exposure to be the first hospital admission during the study period. For patients with transfers between ICUs (i.e., within the same or different hospitals), we created an ICU episode of care using unique patient identifiers. To focus on individuals admitted to the highest-intensity ICUs, we excluded patients whose ICU care involved only an admission to a step-down and/or intermediate care ICU, coronary care unit, or cardiac surgery ICU. Patients who were admitted to both high-intensity and low-intensity ICUs during the index admission were included in the study. We also created a comparator group of patients who were hospitalized during the study period but who were not admitted to an ICU. To identify sicker patients, the non-ICU group was restricted to nonobstetric admissions and patients with a hospital length of stay >2 days. The final study cohorts included 420,187 ICU and 1,603,154 non-ICU patients who survived their index hospitalization (Additional file 1: Figure S1).

To explore postdischarge use of health services, these cohorts were linked to the following health administrative databases using unique encrypted health card numbers: (1) the Ontario Health Insurance Plan database, which contains billing claims for physician services; (2) the National Ambulatory Care Reporting System database, which includes information on all emergency department visits; (3) the Home Care Reporting System database, for data on home care services; (4) the Client Profile Database, which contains data on all applications and admissions to long-term care facilities in Ontario; (5) the National Rehabilitation Reporting System database, which includes information on inpatient rehabilitation services; and (6) the Ontario Registered Persons Database, which includes demographic and vital status (mortality) information on all Ontarians. These datasets are housed at the Institute for Clinical and Evaluative Sciences, which maintains linkable health-related administrative data on all residents of Ontario from 1992 and have been validated and are regularly used for research [18, 19]. Follow-up data were available for a minimum of 1 year and a maximum of 11 years after hospital discharge. Studies in other patient populations suggest that loss to follow-up due to migration out of the province would be minimal (<3 %), with more recent population census data suggesting that less than 7 % of all Ontarians migrated out of the province over a 5-year period [20, 21].

### Outcome measures

The primary outcome was mortality up to 5 years following discharge from the index hospital admission. Secondary outcomes were indicators of health service utilization assessed at 30 days, 6 months, 1 year, 3 years, and 5 years after discharge, including the following: subsequent hospitalizations, including admission to any ICU; emergency department visits; home care visits; inpatient rehabilitative services; and admissions to a long-term care facility. Use of long-term care services included both new admissions and readmissions to these facilities. We also report ICU and hospital mortality during the index hospitalization.

### Patient characteristics and ICU exposures

The demographic characteristics of patients were derived from the DAD and included age (categorized as 18–24 years, 25–34 years, 35–44 years, 45–54 years, 55–64 years, 65–74 years, 75–84 years, and 85 years and older), sex, diagnoses (based on ICD-10 codes for the most responsible diagnoses during the index admission), comorbidities (defined on the basis of the Charlson-Deyo comorbidity index [22–24]), location of patient residence (urban or rural), and income (based on median neighborhood income quintile). For the ICU cohort, we defined clinical variables during the index ICU admission, including the number of episodes of mechanical ventilation, cumulative days in the ICU (length of stay), use of surgical and percutaneously placed endoscopic gastrostomy tube, and tracheostomy.

### Statistical analyses

We compared the patterns of mortality for the ICU and non-ICU survivors across all patients and strata defined by age, sex, and discharge disposition. For the cohort of patients admitted to an ICU, we also stratified by procedures performed during their index ICU admission, including mechanical ventilation, percutaneous gastrostomy tube insertion, and tracheostomy tube insertion. Univariate comparisons were performed using Student’s *t* test or the Wilcoxon rank-sum test for continuous variables, as appropriate, and the χ^2^ statistic for categorical variables. Kaplan-Meier survival curves were generated to describe cumulative survival and time to healthcare resource use following hospital discharge for each patient group. To identify factors associated with mortality following critical illness, Cox proportional hazards models were fitted to the data to estimate the risk of mortality following hospital discharge. These models were restricted to the subgroup of ICU patients and adjusted for patient-level factors, including sex, comorbidities, income quintile, ICU length of stay during index hospitalization, procedures performed during the index ICU admission (mechanical ventilation, percutaneous gastrostomy tube, tracheostomy), discharge disposition (home, home with services, and long-term care), and location of residence.

### Ethics and consent

The institutional research ethics board at Sunnybrook Health Sciences Centre approved the study, and it determined that informed consent was not required.

## Results

The demographic and clinical characteristics of patients during index hospital admissions are detailed in Tables 1 and 2. Patients admitted to an ICU were older [mean age (standard deviation) 63.3 (17.1) vs 60.2 (18.8) years; *p* < 0.001] and were more likely to be male (56.5 % vs 44.5 %; *p* < 0.0001) than non-ICU patients. Myocardial infarction, trauma, and cancer were the most common diagnoses among ICU patients. In-hospital mortality was 16 % among ICU patients and 3.3 % among non-ICU patients (*p* < 0.0001). Patients in the ICU cohort who required more ICU interventions, including mechanical ventilation and tracheostomy, had the highest in-hospital mortality (Additional file 1: Table S1).

**Table 1:** Demographics of ICU and non-ICU patients during index hospital admission^a^

Characteristics	Hospitalizations with ICU admission (*n* = 500,124)	Hospitalizations without ICU admission (*n* = 1,657,940)
Female sex	43.5	55.5
Mean age, years (SD)	63.3 (17.1)	60.2 (18.8)
Age group, years		
18–24	3.0	4.2
25–34	4.3	6.2
35–44	7.4	11.6
45–54	13.6	16.3
55–64	18.7	17.3
65–74	22.4	17.4
75–84	22.6	17.5
≥ 85	7.9	9.5
Charlson score		
0	27.4	58.5
1–2	41.0	29.0
≥3	31.6	12.5
Diagnoses (most responsible)		
Myocardial infarction	25.9	6.8
Trauma	11.8	10.5
Cancer/neoplasm	11.4	13.6
Pneumonia and other infections	5.5	4.1
COPD	3.0	2.2
Peripheral vascular disease	2.8	0.4
Congestive heart failure	2.7	1.4
Musculoskeletal system disorders	2.6	11.7
Diabetes	1.7	1.0
Liver disease	0.5	0.3
Renal disease	0.2	0.1
Dementia	0.1	0.5
Other digestive system	8.4	16.2
Other circulatory system	6.3	2.0
Other respiratory system	4.3	1.6
Other genitourinary	2.0	10.0
Other endocrine	0.9	1.5
Other	10.1	16.2
Income quintile		
Quintile 1 (lowest)	22.9	21.0
Quintile 2	21.1	20.7
Quintile 3	19.3	19.5
Quintile 4	18.8	19.3
Quintile 5 (highest)	17.3	19.2
Rural residence	18.3	14.7
Hospital length of stay, days, median (IQR)	9 (5, 17)	5 (4, 8)
Hospital mortality	16.0	3.3

*COPD* chronic obstructive pulmonary disease, *ICU* intensive care unit, *IQR* interquartile range

Data are presented as proportions (%) unless otherwise stated. Income quintile does not add to 100 % due to missing values for 9940 patients

^a^ *p* < 0.001 for all comparisons

**Table 2:** Demographic and clinical characteristics and outcomes following an index ICU admission in Ontario, 1 April 2002 to 31 March 2012, stratified by survival^a^

Characteristic	Nonsurvivors (*n* = 79,937)	Survivors (*n* = 420,187)
Female sex	44.9	43.3
Age group, years		
18–24	0.84	3.4
25–34	1.5	4.9
35–44	3.3	8.2
45–54	8.2	14.6
55–64	14.1	19.6
65–74	23.0	22.3
75–84	33.3	20.5
≥85	15.8	6.4
Charlson score		
0	11.0	30.5
1–2	36.0	41.9
≥3	53.0	27.6
Most responsible diagnosis		
Myocardial infraction	19.6	27.1
Trauma	8.7	12.3
Cancer/neoplasm	11.3	11.4
Pneumonia and other infections	12.6	4.1
COPD	4.0	2.8
Peripheral vascular disease	2.3	2.9
Congestive heart failure	3.5	2.5
Musculoskeletal system disorders	1.0	2.9
Diabetes	1.0	1.8
Liver disease	1.1	0.4
Renal disease	0.3	0.1
Dementia	0.1	0.1
Other digestive system	9.4	8.2
Other circulatory system	5.2	6.5
Other respiratory system	8.8	3.4
Other genitourinary	2.0	2.0
Other endocrine	0.6	1.0
Other	8.6	10.4
Income quintile		
Quintile 1 (lowest)	24.8	22.6
Quintile 2	21.9	21.0
Quintile 3	18.5	19.4
Quintile 4	17.7	19.0
Quintile 5 (highest)	16.6	17.5
Rural residence	13.3	19.3
Procedures		
Mechanically ventilated	70.4	33.4
Pulmonary artery catheter	8.3	10.7
Bronchoscopy	16.2	8.0
Thoracostomy tube insertion	6.7	4.5
Dialysis	10.9	2.9
Percutaneous feeding tube insertion	7.5	3.5
Tracheostomy tube insertion	6.6	2.7
Intracranial pressure monitoring	1.9	1.1
Transvenous pacemaker insertion	1.2	0.8
Intraaortic balloon counterpulsation device insertion	1.6	0.7
Complications and outcomes		
Pneumonia	5.8	2.4
Surgical site infection	3.4	2.6
Blood infection	7.1	1.2
*Clostridium difficile* infection	2.0	0.9
Duration of ventilation, days, median (IQR)	4 (2, 9)	2 (1, 5)
Cumulative ICU days		
0 to <3 days	48.9	58.5
3 to <5 days	13.3	18.1
5 to <7 days	8.5	8.4
7 to <14 days	15.0	9.5
14 to <60 days	12.8	5.1
60 to <120 days	1.2	0.3
≥120 days	0.4	0.1
Hospital length of stay, days, median (IQR)	9 (3, 20)	9 (5, 17)

*COPD* chronic obstructive pulmonary disease, *ICU* intensive care unit, *IQR* interquartile range

Note income quintiles do not add to 100 % due to missing values for 2733 patients. Data are presented as proportions (%) unless otherwise stated. ICU days could not be determined for 15 patients

^a^ *p* < 0.001 for all comparisons

### Mortality following discharge

**Fig. 1:**
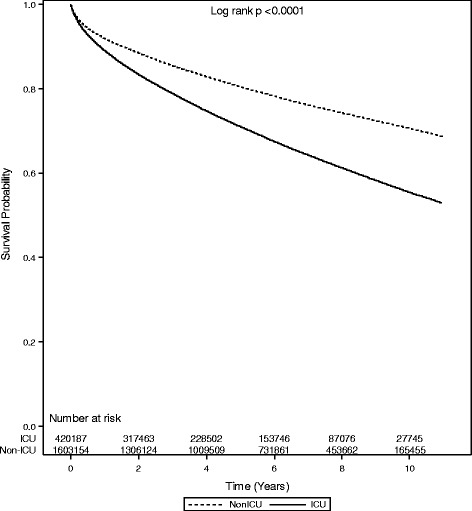
Cumulative survival of patients discharged from an acute hospitalization in Ontario, 1 April 2002 to 31 March 2012, stratified by intensive care unit (ICU) admission status

A total of 420,187 ICU (84 %) and 1,603,154 (97 %) non-ICU patients survived to hospital discharge. During the follow-up period (median 5.3 years [interquartile range 2.5, 8.2]), there were 136,154 (32 %) posthospitalization deaths among the ICU patients and 358,624 (22 %) deaths among the non-ICU patients. For both the ICU and non-ICU groups, approximately one-fourth of deaths occurred within the first 6 months after discharge (Fig. 1, Table 3, and Additional file 1: Table S2). The pattern of higher deaths among ICU patients was consistent in sensitivity analysis that excluded patients who died within the first 6 months after discharge (Additional file 1: Figure S2). Among the ICU patients, mortality was higher among patients discharged to long-term care, and increased with longer ICU length of stay (Table 3). At 1 year, mortality among those aged 75–84 years was 17.5 %, compared with 6.8 % among younger (<75 years) survivors of critical illness (*p* < 0.0001 for age group comparison). This pattern of increasing mortality with increasing age was consistent across all time points assessed (Table 3). In a multivariable analysis examining predictors of mortality among ICU survivors, patients who were between 65 and 74 years of age had a sevenfold increased hazard of death during follow-up compared with patients younger than 25 years of age (hazard ratio [HR] 7.39, 95 % confidence interval [CI] 6.79–8.04), while patients aged 85 years and older had an 18-fold higher hazard of death (HR 18.08, 95 % CI 16.60–19.68) (Table 4, Additional file 1: Table S3).

**Table 3:** Cumulative mortality after hospital discharge among ICU patients

Characteristics	30 days (*n* = 411,345)	6 months (*n* = 389,896)	1 year (*n* = 373,822)	3 years (*n* = 270,768)	5 years (*n* = 189,996)	Raw mortality postdischarge
Mortality	2.2	7.2	11.1	21.2	29.0	32.4
Mortality by age, years						
18–24	0.3	0.8	1.3	2.8	3.6	3.9
25–34	0.4	1.4	2.3	4.4	5.8	6.5
35–44	0.6	2.4	3.8	7.2	9.8	11.1
45–54	1	3.6	5.9	11.3	15	16.6
55–64	1.4	5.2	8.2	15.9	21.7	24.3
65–74	2.2	7.9	12.2	23.3	32.3	37.2
75–84	3.6	11.7	17.5	34.1	47.5	53.9
≥85	6.7	19.4	28.1	51.8	68.7	70.0
Mortality by sex						
Male	2.1	7.0	10.8	20.7	28.2	31.6
Female	2.2	7.5	11.4	21.9	30.1	33.5
Mortality by resource use						
Mechanical ventilation						
No	2.0	7.1	10.9	21.1	28.9	32.8
Yes	2.4	7.6	11.4	21.4	29.2	31.7
Tracheostomy						
No	2.1	7.1	10.9	21.0	28.8	32.2
Yes	3.4	11.5	17.2	30.6	39.2	39.9
Percutaneous feeding tube						
No	2.1	6.9	10.6	20.6	28.4	31.8
Yes	5.0	15.7	23.0	38.6	47.9	47.8
Mortality by cumulative ICU days						
0 to <3 days	1.7	6.1	9.6	18.7	25.8	29.3
3 to <5 days	2.3	7.7	11.8	22.9	31.5	35.0
5 to <7 days	2.7	9.1	13.4	25.3	34.4	37.7
7 to <14 days	3.1	9.7	14.4	26.4	35.7	38.3
14 to <60 days	3.4	10.4	15.1	26.9	35.8	37.6
60 to <120 days	3.6	14.1	19.4	34.7	46.0	45.8
≥120 days	4.3	14.9	22.3	43.1	52.2	50.1
Mortality by discharge disposition						
Home and other	1.0	4.2	7.1	15.1	21.8	26.0
Home with services	2.6	10.6	16.6	31.4	41.6	43.5
Acute care/other facility	4.2	10.3	14.3	24.4	32.8	41.1
Long-term care	7.2	17.8	23.8	39.4	51.3	48.3

*ICU* intensive care unit

Mortality from 30 days to 5 years and number at risk were estimated by Kaplan-Meier survival analysis. *p* < 0.001 by log-rank test for all comparisons. Raw mortality calculated as number of deaths among survivors over total population in each group

**Table 4:** Multivariable analyses of factors associated with postdischarge mortality among ICU survivors

Characteristics	Hazard ratio (95 % CI)
Sex	
Men	Reference
Women	0.91 (0.90–0.92)
Age group, years	
18–24	Reference
25–34	1.57 (1.42-1.73)
35–44	2.48 (2.27–2.71)
45–54	3.62 (3.32–3.94)
55–64	4.99 (4.59–5.43)
65–74	7.39 (6.79–8.04)
75–84	11.30 (10.39–12.29)
≥85	18.08 (16.60–19.68)
Mechanical ventilation	
None	Reference
Yes	0.88 (0.87–0.89)
Tracheostomy	
None	Reference
Yes	1.04 (1.00–1.08)
Percutaneous feeding tube	
None	Reference
Yes	1.32 (1.28–1.35)
Cumulative ICU days	
0 to <3 days	Reference
3 to <5 days	1.04 (1.03–1.05)
5 to <7 days	1.08 (1.06–1.10)
7 to <14 days	1.07 (1.05–1.09)
14 to <60 days	1.00 (0.97–1.02)
60 to <120 days	1.00 (0.91–1.09)
≥120 days	1.20 (1.01–1.42)
Most responsible diagnosis^a^	
Myocardial infarction	Reference
Trauma	1.10 (1.07–1.13)
Cancer/neoplasm	1.47 (1.44–1.51)
Pneumonia and other infections	1.62 (1.58–1.67)
COPD	2.13 (2.07–2.20)
Congestive heart failure	1.72 (1.67–1.77)
Presence of comorbidities^a^	
Myocardial infarction	0.91 (0.90–0.93)
Congestive heart failure	0.64 (0.63–0.65)
Peripheral vascular disease	0.77 (0.75–0.78)
Dementia	0.64 (0.63–0.66)
COPD	0.70 (0.69–0.71)
Moderate/severe liver disease	0.37 (0.36–0.39)
Discharge disposition	
Home and other	Reference
Acute care/other facility	1.43 (1.40–1.47)
Home with services	1.30 (1.29–1.32)
Long-term care	1.83 (1.80–1.86)
Area	
Urban	Reference
Rural	1.00 (0.98–1.01)
Income quintile	
Quintile 1 (lowest)	1.14 (1.12–1.16)
Quintile 2	1.08 (1.06–1.10)
Quintile 3	1.07 (1.05–1.09)
Quintile 4	1.04 (1.03–1.06)
Quintile 5 (highest)	Reference

*CI* confidence interval, *COPD* chronic obstructive pulmonary disease, *ICU* intensive care unit

^a^A full list of the most responsible diagnoses and comorbidities is provided in Additional file 1: Table S3

### Healthcare utilization following discharge

**Fig. 2:**
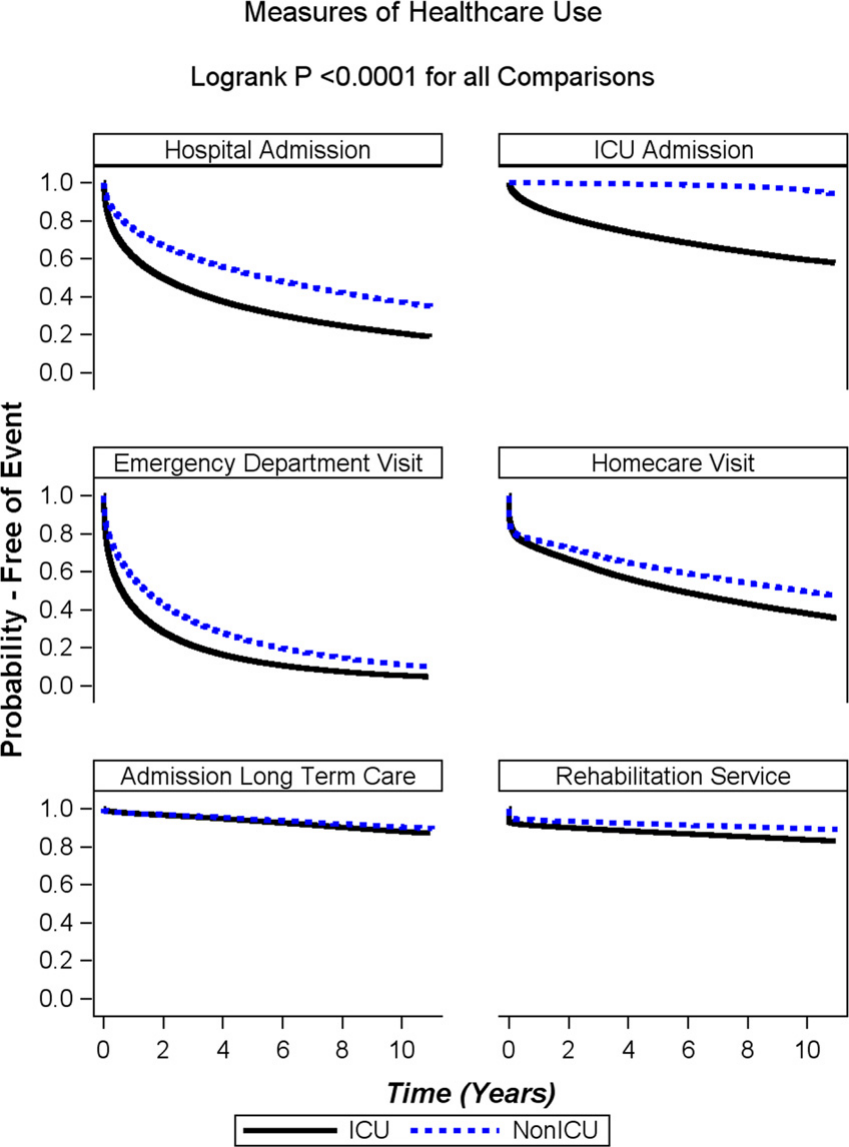
Kaplan-Meier curves for time to health service use following discharge from the hospital, stratified by intensive care unit (ICU) admission status

Emergency department and acute care hospital admissions were common after discharge, with utilization higher among ICU survivors: 84.1 % (emergency department visits) and 65.3 % (hospital readmissions) of ICU survivors experienced one or more of these events during follow-up, compared with 75.8 % and 49.7 % among non-ICU survivors, respectively (*p* < 0.0001 by log-rank test for both comparisons). The majority of hospitalizations occurred within the first year following hospital discharge, a pattern consistent across the two groups (Fig. 2). The primary diagnosis for hospital readmission was the same as the index admission for 27.7 % of patients surviving critical illness. One-fourth of ICU survivors had been readmitted to ICU during the 5 years following the index hospitalization. Compared with non-ICU patients, ICU patients had a statistically significant, but not clinically important, higher estimated probability of being admitted to a long-term care facility at 5 years (6.4 % and 5.5 % for ICU and non-ICU patients, respectively; *p* < 0.001 by log-rank test). Similar findings were noted for use of rehabilitation services.

## Discussion

We studied all acute care hospitalizations that included an ICU admission in Canada’s largest province and spanning an entire decade. We found that most patients who survived to be discharged from the hospital following an ICU admission subsequently required additional healthcare services. In particular, almost half (46 %) were admitted to an emergency department and nearly one-third (29 %) were rehospitalized within the first 6 months following discharge from the hospital. In contrast, postdischarge health service utilization rates were considerably less among patients whose initial hospitalization did not include an ICU admission. Approximately one-third of patients who survived their critical illness died during the follow-up period, with notable differences in mortality by age and need for ICU interventions during the index hospital admission.

Our findings extend prior work in this area that has demonstrated increased utilization of healthcare services following discharge from the hospital after critical illness [3, 6, 7, 9, 15, 25]. Our observation of significantly higher rates of readmission to the hospital among ICU survivors than among non-ICU survivors is consistent with some previous studies but differs from others [7, 12, 15]. In their population-based study, Garland and colleagues reported a 41 % rehospitalization rate among both non-ICU and ICU patients in the year following discharge [15]. In contrast, we observed that at 1 year following discharge, ICU survivors experienced a 60 % higher hospital readmission rate than non-ICU patients (38.4 % vs 23.9 %, respectively). While the explanation for this difference is unclear, it may relate to differences in case mix, patient preferences regarding rehospitalization, and system factors that influence hospital admission decisions. However, our finding of substantial higher use of ICU services among ICU survivors than non-ICU patients is consistent with the report by Garland and colleagues. Whether these increased healthcare needs are related to new care requirements for newly acquired illnesses, exacerbation of the causes underlying the prior critical illness, or lack of access to appropriate follow-up care cannot be determined on the basis of our study. However, our data demonstrating that 28 % of all readmissions were for related diagnoses suggest that worsening of prior illness likely accounts for a substantial proportion of the readmission. The high rate (23 %) of emergency department admissions within 30 days of discharge among ICU survivors suggests that post-ICU care could be improved. Several other reports have described physical, neurological, and psychological impairments among survivors of ICU care, and have advocated a need for improved discharge planning and follow-up care for these individuals [3, 4, 26, 27].

Our finding that nearly one-third of ICU survivors died during the follow-up period aligns with previous work [6, 7, 28]. We found that a significant proportion of these deaths occurred among the elderly, with age being an independent strong predictor of mortality. While these results are not unexpected, that one in five survivors of critical illness aged 85 years or older die within 6 months after hospital discharge suggests that there may be opportunities for informing patient and provider decisions regarding the increased risk of dying after an ICU stay in this subgroup. The observed negative association between days in the ICU and long-term mortality following critical illness has previously been demonstrated [28, 29] and suggests that longer stay may identify a subgroup of patients for whom interventions to reduce poor outcomes after discharge may be targeted. While the most robust studies to date have provided no evidence of the benefit of postdischarge interventions for survivors of critical care, identifying the patient population mostly likely to benefit from these interventions is an important objective [30, 31].

This study has several limitations. First, we did not have access to other important predictors of outcome, such as preexisting or post-ICU frailty markers, or physical examination–based markers of muscle strength or conditioning, or measures of functional independence. However, we described and accounted for age and preexisting comorbidities, which have been shown to be more influential in predicting long-term mortality [28, 32]. Although some of these factors may explain more specifically some of the influence of age that we observed, our findings indicate that, in the absence of frailty and functional measures, age is a strong and intuitive predictor of outcomes for many patients and a useful trigger for clinicians to ensure that patients and families are well-informed about the benefits and potential risks of critical illness and critical care. Second, our use of administrative data limits our ability to identify some potentially important risk factors for health service use following critical illness, including availability of family and social supports and patient preferences regarding care. Third, our estimates of home care utilization after hospital discharge address services covered by public funds and do not include services paid for through private insurance or out of pocket. However, there is no evidence to suggest that availability of private coverage differs for ICU and non-ICU patients; therefore, this should have had minimal impact on our comparison of home care service use. Fourth, our study is unable to establish causal factors for the observed increased healthcare utilization following ICU discharge, and this is an important focus for future research. These considerations notwithstanding, our population-based cohort, linkages across several databases to describe healthcare use and mortality, and inclusion of a breadth of ICU survivors are important strengths of the study.

## Conclusions

We found that a majority of survivors of critical illness require additional emergency department, acute care, and long-term care services. Furthermore, the use of these services began in the period immediately after discharge. Mortality is high among elderly critical illness survivors, especially among those requiring the greatest resources while in the hospital. These findings provide data for more informed goals-of-care discussions and may help target post–ICU discharge services for these high-risk groups.

## Key messages

- In this largest population-based cohort study done to date, we found that survivors of critical illness tended to have higher healthcare resource utilization, including hospital and ICU admissions, than other hospitalized patients.
- Among ICU patients, healthcare resource use and mortality following discharge were highest among older patients and those who required more ICU procedures during the index admission.

## Additional file

Additional file 1. Supplementary Figures and Tables. (DOC 204 kb)

## Abbreviations

CIHI: Canadian Institute for Health Information
CI: confidence interval
COPD: chronic obstructive pulmonary disease
DAD: Discharge Abstract Database
HR: hazard ratio
ICD-10: International Classification of Diseases, Tenth Revision
ICU: intensive care unit
IQR: interquartile range
