# Supplementary material for: Long-term outcomes and healthcare utilization following critical illness – a population-based study
Source: Crit Care. 2016 Mar 31;20:76. doi: 10.1186/s13054-016-1248-y (PMC4818427; doi:10.1186/s13054-016-1248-y)
Supplement: Additional file 1. — Supplementary Figures and Tables. (DOC 204 kb) [file 13054_2016_1248_MOESM1_ESM.doc]

**Supplementary Tables and Figures**

Table S1: Hospital outcome among patients admitted to an ICU

| Characteristics  (N=500,124) | ICU Mortality  (%) | Hospital Mortality  (%) |
| --- | --- | --- |
| All | 10.7 | 16.0 |
| Age |  |  |
| 18 - 24 | 3.8 | 4.4 |
| 25 - 34 | 4.5 | 5.5 |
| 35 - 44 | 5.7 | 7.1 |
| 45 - 54 | 7.4 | 9.7 |
| 55 - 64 | 8.8 | 12 |
| 65 - 74 | 11.3 | 16.4 |
| 75 - 84 | 14.8 | 23.6 |
| ≥ 85 | 17.9 | 31.9 |
| Sex |  |  |
| Men | 10.45 | 15.6 |
| Women | 10.97 | 16.5 |
| Mechanical Ventilation | |  |
| No | 3.7 | 7.8 |
| Yes | 21.5 | 28.6 |
| Tracheostomy |  |  |
| No | 10.3 | 15.4 |
| Yes | 21.3 | 32.1 |
| Percutaneous  Feeding tube | |  |
| No | 10.5 | 15.4 |
| Yes | 15.7 | 28.9 |

Table S2: Cumulative mortality after discharge among non-ICU patients

|  |  |  |  |  |  |  |
| --- | --- | --- | --- | --- | --- | --- |
| Number at risk  Characteristics | 30-days  (1,574,564) | 6-months  (1,512,648) | 1 year  (1,473,282) | 3 years  (1,154,057) | 5 years  (869,149) | Raw mortality post discharge |
| Mortality | 1.8 | 5.7 | 8.1 | 14.5 | 19.6 | 22.4 |
| Mortality by Age |  |  |  |  |  |  |
| 18 - 24 | 0.0 | 0.1 | 0.3 | 0.6 | 0.9 | 1.1 |
| 25 - 34 | 0.1 | 0.3 | 0.5 | 1.0 | 1.4 | 1.7 |
| 35 - 44 | 0.2 | 0.8 | 1.2 | 2.1 | 2.8 | 3.4 |
| 45 - 54 | 0.5 | 1.9 | 2.8 | 4.8 | 6.1 | 6.9 |
| 55 - 64 | 1.1 | 3.7 | 5.4 | 8.9 | 11.3 | 12.6 |
| 65 - 74 | 1.9 | 6.3 | 9.0 | 15.4 | 20.4 | 24.0 |
| 75 - 84 | 3.5 | 10.7 | 15.3 | 28.3 | 39.7 | 47.1 |
| ≥ 85 | 7.1 | 19.4 | 27.3 | 50.5 | 68.1 | 72.8 |
| Mortality by Sex |  |  |  |  |  |  |
| Men | 2.0 | 6.1 | 8.8 | 15.3 | 20.2 | 22.5 |
| Women | 1.7 | 5.3 | 7.6 | 13.9 | 19.1 | 22.3 |
| Mortality by Discharge Disposition | |  |  |  |  |  |
| Home and other | 0.7 | 3.0 | 4.7 | 9.2 | 12.8 | 15.4 |
| Home care | 2.9 | 9.8 | 13.7 | 23.6 | 31.1 | 33.0 |
| Acute care/other facility | 4.0 | 9.2 | 12.3 | 21.3 | 29.1 | 41.0 |
| Long term care | 9.1 | 20.1 | 26.2 | 43.0 | 56.1 | 56.9 |

*Mortality 30 days to 5 years and number at risk were estimated from Kaplan-Meier survival analysis.*

*Raw mortality calculated as number of deaths among survivors over total population in each group.*

Table S3: Multivariable analyses of factors associated with post-discharge mortality among ICU survivors

| **Characteristics** | **Hazard Ratio 95% Confidence Interval** |
| --- | --- |
| Sex |  |
| Men | Reference |
| Women | 0.91 (0.90, 0.92) |
| Age Group |  |
| 18 - 24 | Reference |
| 25 - 34 | 1.57 (1.42,1.73) |
| 35 - 44 | 2.48 (2.27, 2.71) |
| 45 - 54 | 3.62 (3.32, 3.94) |
| 55 - 64 | 4.99 (4.59, 5.43) |
| 65 - 74 | 7.39 (6.79, 8.04) |
| 75 - 84 | 11.30 (10.39, 12.29) |
| ≥ 85 | 18.08 (16.60, 19.68) |
| Mechanical Ventilation |  |
| None | Reference |
| Yes | 0.88 (0.87, 0.89) |
| Tracheostomy |  |
| None | Reference |
| Yes | 1.04 (1.00, 1.08) |
| Percutaneous Feeding Tube |  |
| None | Reference |
| Yes | 1.32 (1.28, 1.35) |
| Cumulative ICU days |  |
| 0 to <3 days | Reference |
| 3 to <5 days | 1.04 (1.03, 1.05) |
| 5 to <7 days | 1.08 (1.06, 1.10) |
| 7 to <14 days | 1.07 (1.05, 1.09) |
| 14 to <60 days | 1.00 (0.97, 1.02) |
| 60 to <120 days | 1.00 (0.91, 1.09) |
| > 120 days | 1.20 (1.01, 1.42) |
| Most Responsible Diagnosis* |  |
| Myocardial infarction | Reference |
| Trauma | 1.10 (1.07, 1.13) |
| Cancer/neoplasm | 1.47 (1.44, 1.51) |
| Pneumonia and other infections | 1.62 (1.58, 1.67) |
| COPD | 2.13 (2.07, 2.20) |
| Congestive heart failure | 1.72 (1.67, 1.77) |
| Diabetes | 1.56 (1.50, 1.64) |
| Dementia | 1.42 (1.23, 1.64) |
| Liver disease | 1.57 (1.45, 1.70) |
| Renal Disease | 1.95 (1.75, 2.18) |
| Peripheral vascular disease | 0.96 (0.93, 1.00) |
| Musculoskeletal system disorders | 0.80 (0.77, 0.83) |

Table S3 (cont’d): Multivariable analyses of factors associated with post-discharge mortality among ICU survivors

| **Characteristics** | **Hazard Ratio 95% Confidence Interval** |
| --- | --- |
| Most Responsible Diagnosis |  |
| Other respiratory | 1.97 (1.91, 2.03) |
| Other endocrine | 1.50 (1.41, 1.59) |
| Other genitourinary | 1.42 (1.37, 1.48) |
| Other digestive | 1.29 (1.26, 1.32) |
| Other circulatory | 1.22 (1.19, 1.25) |
| Other | 1.34 (1.31, 1.37) |
| Presence of Comorbidities |  |
| Myocardial infarction | 0.91 (0.90, 0.93) |
| Congestive heart failure | 0.64 (0.63, 0.65) |
| Peripheral vascular disease | 0.77 (0.75, 0.78) |
| Dementia | 0.64 (0.63, 0.66) |
| COPD | 0.70 (0.69, 0.71) |
| Moderate/severe liver disease | 0.37 (0.36, 0.39) |
| Mild liver disease | 0.63 (0.60, 0.65) |
| Cerebrovascular disease | 0.84 (0.82, 0.85) |
| Connective tissue/rheumatic disease | 0.71 (0.68, 0.73) |
| Peptic ulcer disease | 0.88 (0.86, 0.90) |
| Diabetes without complications | 0.84 (0.83, 0.85) |
| Diabetes with complications | 0.83 (0.81, 0.84) |
| Hemiplegia/paraplegia | 0.77 (0.75, 0.80) |
| Renal disease | 0.65 (0.64, 0.67) |
| Primary cancer | 0.54 (0.53, 0.55) |
| Metastatic cancer | 0.21 (0.20, 0.21) |
| HIV/AIDS | 0.46 (0.40, 0.52) |
| Discharge Disposition |  |
| Home and other | Reference |
| Acute care/other facility | 1.43 (1.40, 1.47) |
| Home with services | 1.30 (1.29, 1.32) |
| Long term care | 1.83 (1.80, 1.86) |
| Area |  |
| Urban | Reference |
| Rural | 1.00 (0.98, 1.01) |
| Income Quintile |  |
| Quintile 1 (lowest) | 1.14 (1.12, 1.16) |
| Quintile 2 | 1.08 (1.06, 1.10) |
| Quintile 3 | 1.07 (1.05, 1.09) |
| Quintile 4 | 1.04 (1.03, 1.06) |
| Quintile 5 (highest) | Reference |

Figure S1: Flowchart of patient cohort creation

Hospital separations between April 1, 2002 and March 31, 2012

N= 5,473,411

Included an ICU Admission

N= 982,566

Patients with an index ICU admission between April 1, 2002 and March 31, 2012

N =978,810

Exclude ICU admission prior to April 1, 2002

N= 3756

ICU patients

N =500,124

Exclude

1. Patients < 18 years (N=212,257)
2. Patients without an admission to a high intensity ICU (N=266,388)
3. Patients with death date data entry error (N=41)

Did not include an ICU Admission

N= 4,490,845

Non-ICU patients

N = 1,657,940

Exclude

1. Patients < 18 years (N=1,418,502)
2. Obstetrical admissions (N=826,341)
3. Length of hospital stay < 2 days (N=587,956)
4. Patients with death date data entry error (N=106)

Cohort of non-ICU survivors

N = 1,603,154

Cohort of ICU survivors

N =420,187

Died in hospital

N= 54,786

Died in hospital

N= 79,937

Figure S2: Deaths following discharge (excludes deaths within the first 6-months of discharge)
